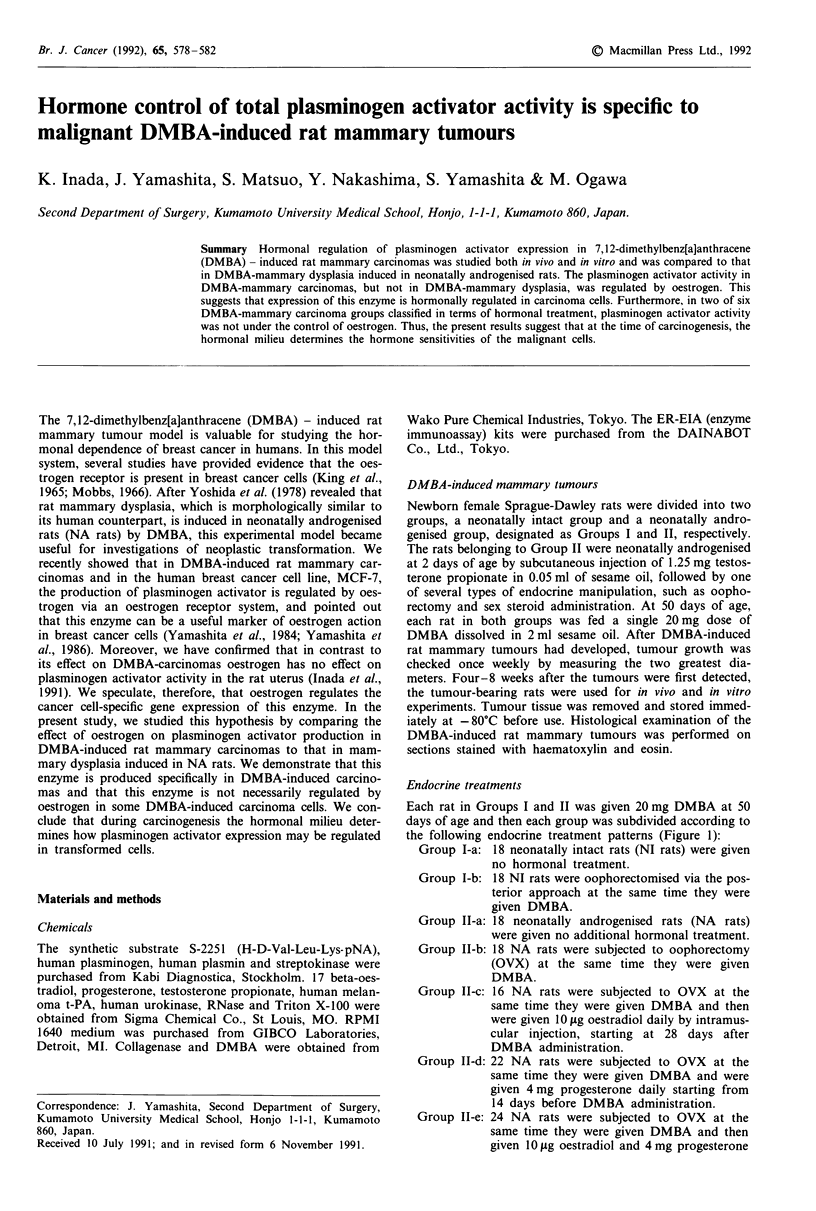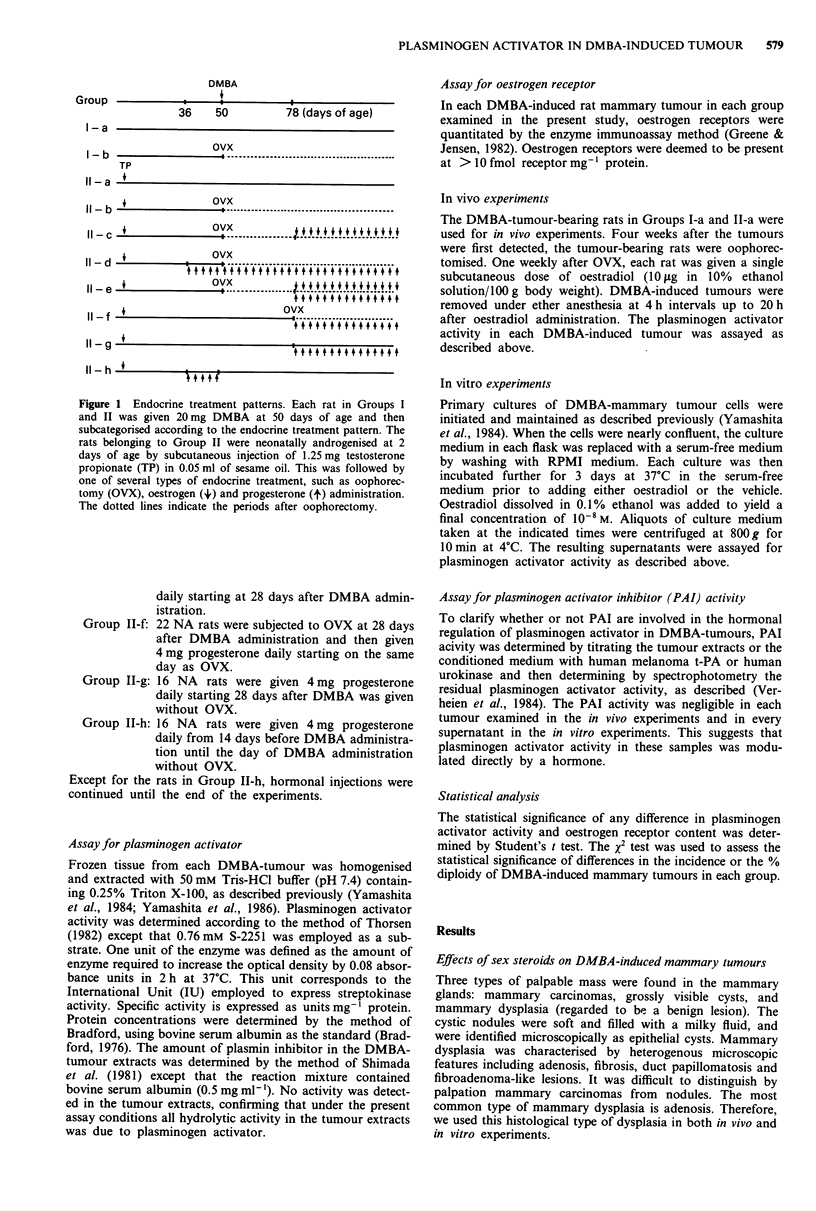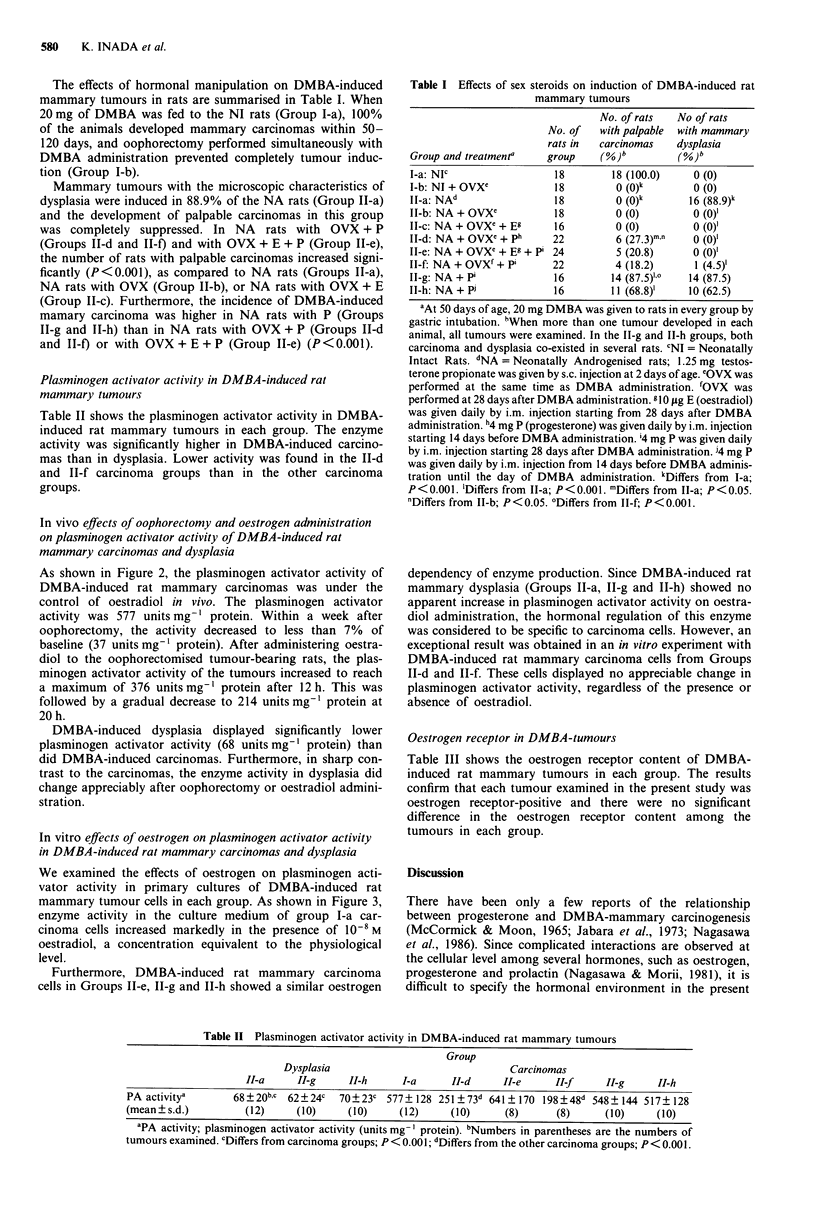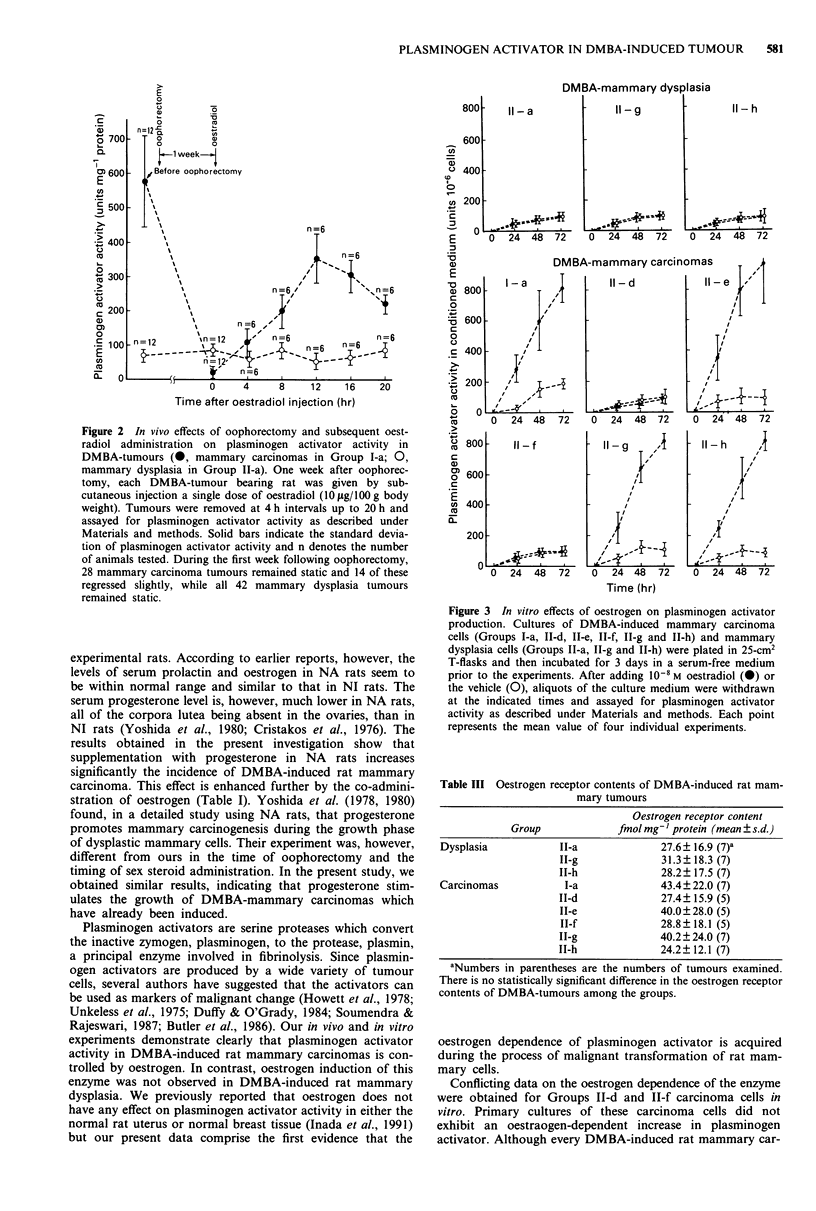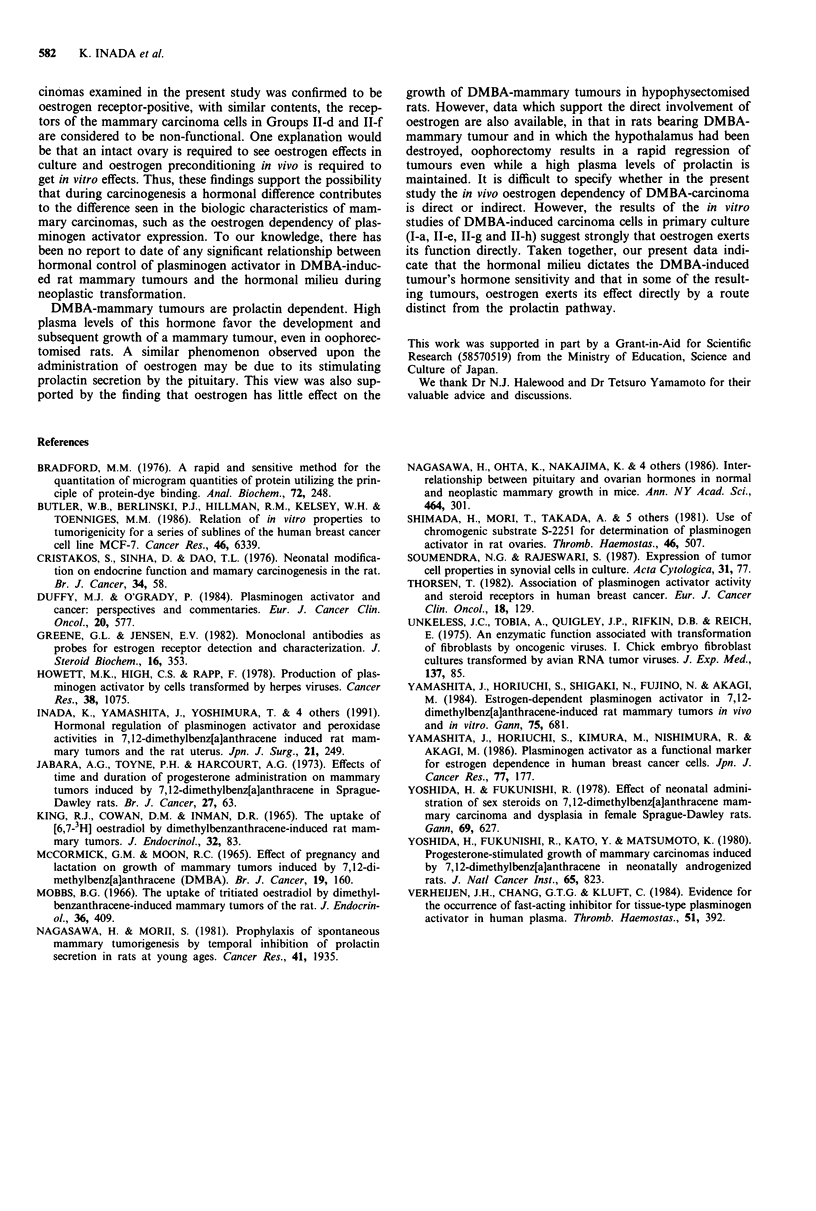# Hormone control of total plasminogen activator activity is specific to malignant DMBA-induced rat mammary tumours.

**DOI:** 10.1038/bjc.1992.117

**Published:** 1992-04

**Authors:** K. Inada, J. Yamashita, S. Matsuo, Y. Nakashima, S. Yamashita, M. Ogawa

**Affiliations:** Second Department of Surgery, Kumamoto University Medical School, Japan.

## Abstract

Hormonal regulation of plasminogen activator expression in 7,12-dimethylbenz[a]anthracene (DMBA)--induced rat mammary carcinomas was studied both in vivo and in vitro and was compared to that in DMBA-mammary dysplasia induced in neonatally androgenised rats. The plasminogen activator activity in DMBA-mammary carcinomas, but not in DMBA-mammary dysplasia, was regulated by oestrogen. This suggests that expression of this enzyme is hormonally regulated in carcinoma cells. Furthermore, in two of six DMBA-mammary carcinoma groups classified in terms of hormonal treatment, plasminogen activator activity was not under the control of oestrogen. Thus, the present results suggest that at the time of carcinogenesis, the hormonal milieu determines the hormone sensitivities of the malignant cells.


Br. J. Cancer (1992), 65, 578–582 © Macmillan Press Ltd., 1992

### Hormone control of total plasminogen activator activity is specific to malignant DMBA-induced rat mammary tumours

K. Inada, J. Yamashita, S. Matsuo, Y. Nakashima, S. Yamashita & M. Ogawa

*Second Department of Surgery, Kumamoto University Medical School, Honjo, 1-1-1, Kumamoto 860, Japan.*

**Summary** Hormonal regulation of plasminogen activator expression in 7,12-dimethylbenz[a]anthracene (DMBA) – induced rat mammary carcinomas was studied both *in vivo* and *in vitro* and was compared to that in DMBA-mammary dysplasia induced in neonatally androgenised rats. The plasminogen activator activity in DMBA-mammary carcinomas, but not in DMBA-mammary dysplasia, was regulated by oestrogen. This suggests that expression of this enzyme is hormonally regulated in carcinoma cells. Furthermore, in two of six DMBA-mammary carcinoma groups classified in terms of hormonal treatment, plasminogen activator activity was not under the control of oestrogen. Thus, the present results suggest that at the time of carcinogenesis, the hormonal milieu determines the hormone sensitivities of the malignant cells.

The 7,12-dimethylbenz[a]anthracene (DMBA) – induced rat mammary tumour model is valuable for studying the hormonal dependence of breast cancer in humans. In this model system, several studies have provided evidence that the oestrogen receptor is present in breast cancer cells (King *et al.*, 1965; Mobbs, 1966). After Yoshida *et al.* (1978) revealed that rat mammary dysplasia, which is morphologically similar to its human counterpart, is induced in neonatally androgenised rats (NA rats) by DMBA, this experimental model became useful for investigations of neoplastic transformation. We recently showed that in DMBA-induced rat mammary carcinomas and in the human breast cancer cell line, MCF-7, the production of plasminogen activator is regulated by oestrogen via an oestrogen receptor system, and pointed out that this enzyme can be a useful marker of oestrogen action in breast cancer cells (Yamashita *et al.*, 1984; Yamashita *et al.*, 1986). Moreover, we have confirmed that in contrast to its effect on DMBA-carcinomas oestrogen has no effect on plasminogen activator activity in the rat uterus (Inada *et al.*, 1991). We speculate, therefore, that oestrogen regulates the cancer cell-specific gene expression of this enzyme. In the present study, we studied this hypothesis by comparing the effect of oestrogen on plasminogen activator production in DMBA-induced rat mammary carcinomas to that in mammary dysplasia induced in NA rats. We demonstrate that this enzyme is produced specifically in DMBA-induced carcinomas and that this enzyme is not necessarily regulated by oestrogen in some DMBA-induced carcinoma cells. We conclude that during carcinogenesis the hormonal milieu determines how plasminogen activator expression may be regulated in transformed cells.

### Materials and methods

### *Chemicals*

The synthetic substrate S-2251 (H-D-Val-Leu-Lys-pNA), human plasminogen, human plasmin and streptokinase were purchased from Kabi Diagnostica, Stockholm. 17 beta-oestradiol, progesterone, testosterone propionate, human melanoma t-PA, human urokinase, RNase and Triton X-100 were obtained from Sigma Chemical Co., St Louis, MO. RPMI 1640 medium was purchased from GIBCO Laboratories, Detroit, MI. Collagenase and DMBA were obtained from Wako Pure Chemical Industries, Tokyo. The ER-EIA (enzyme immunoassay) kits were purchased from the DAINABOT Co., Ltd., Tokyo.

Correspondence: J. Yamashita, Second Department of Surgery, Kumamoto University Medical School, Honjo 1-1-1, Kumamoto 860, Japan.
Received 10 July 1991; and in revised form 6 November 1991.

### *DMBA-induced mammary tumours*

Newborn female Sprague-Dawley rats were divided into two groups, a neonatally intact group and a neonatally androgenised group, designated as Groups I and II, respectively. The rats belonging to Group II were neonatally androgenised at 2 days of age by subcutaneous injection of 1.25 mg testosterone propionate in 0.05 ml of sesame oil, followed by one of several types of endocrine manipulation, such as oophorectomy and sex steroid administration. At 50 days of age, each rat in both groups was fed a single 20 mg dose of DMBA dissolved in 2 ml sesame oil. After DMBA-induced rat mammary tumours had developed, tumour growth was checked once weekly by measuring the two greatest diameters. Four–8 weeks after the tumours were first detected, the tumour-bearing rats were used for *in vivo* and *in vitro* experiments. Tumour tissue was removed and stored immediately at −80°C before use. Histological examination of the DMBA-induced rat mammary tumours was performed on sections stained with haematoxylin and eosin.

### *Endocrine treatments*

Each rat in Groups I and II was given 20 mg DMBA at 50 days of age and then each group was subdivided according to the following endocrine treatment patterns (Figure 1):

Group I-a: 18 neonatally intact rats (NI rats) were given no hormonal treatment.

Group I-b: 18 NI rats were oophorectomised via the posterior approach at the same time they were given DMBA.

Group II-a: 18 neonatally androgenised rats (NA rats) were given no additional hormonal treatment.

Group II-b: 18 NA rats were subjected to oophorectomy (OVX) at the same time they were given DMBA.

Group II-c: 16 NA rats were subjected to OVX at the same time they were given DMBA and then were given 10 μg oestradiol daily by intramuscular injection, starting at 28 days after DMBA administration.

Group II-d: 22 NA rats were subjected to OVX at the same time they were given DMBA and were given 4 mg progesterone daily starting from 14 days before DMBA administration.

Group II-e: 24 NA rats were subjected to OVX at the same time they were given DMBA and then given 10 μg oestradiol and 4 mg progesterone

**Figure 1** Endocrine treatment patterns. Each rat in Groups I and II was given 20 mg DMBA at 50 days of age and then subcategorised according to the endocrine treatment pattern. The rats belonging to Group II were neonatally androgenised at 2 days of age by subcutaneous injection of 1.25 mg testosterone propionate (TP) in 0.05 ml of sesame oil. This was followed by one of several types of endocrine treatment, such as oophorectomy (OVX), oestrogen (↓) and progesterone (↑) administration. The dotted lines indicate the periods after oophorectomy.

daily starting at 28 days after DMBA administration.

Group II-f: 22 NA rats were subjected to OVX at 28 days after DMBA administration and then given 4 mg progesterone daily starting on the same day as OVX.

Group II-g: 16 NA rats were given 4 mg progesterone daily starting 28 days after DMBA was given without OVX.

Group II-h: 16 NA rats were given 4 mg progesterone daily from 14 days before DMBA administration until the day of DMBA administration without OVX.

Except for the rats in Group II-h, hormonal injections were continued until the end of the experiments.

*Assay for plasminogen activator*

Frozen tissue from each DMBA-tumour was homogenised and extracted with 50 mM Tris-HCl buffer (pH 7.4) containing 0.25% Triton X-100, as described previously (Yamashita *et al.*, 1984; Yamashita *et al.*, 1986). Plasminogen activator activity was determined according to the method of Thorsen (1982) except that 0.76 mM S-2251 was employed as a substrate. One unit of the enzyme was defined as the amount of enzyme required to increase the optical density by 0.08 absorbance units in 2 h at 37°C. This unit corresponds to the International Unit (IU) employed to express streptokinase activity. Specific activity is expressed as units $mg^{-1}$ protein. Protein concentrations were determined by the method of Bradford, using bovine serum albumin as the standard (Bradford, 1976). The amount of plasmin inhibitor in the DMBA-tumour extracts was determined by the method of Shimada *et al.* (1981) except that the reaction mixture contained bovine serum albumin (0.5 mg $ml^{-1}$). No activity was detected in the tumour extracts, confirming that under the present assay conditions all hydrolytic activity in the tumour extracts was due to plasminogen activator.

*Assay for oestrogen receptor*

In each DMBA-induced rat mammary tumour in each group examined in the present study, oestrogen receptors were quantitated by the enzyme immunoassay method (Greene & Jensen, 1982). Oestrogen receptors were deemed to be present at $>10$ fmol receptor $mg^{-1}$ protein.

In vivo *experiments*

The DMBA-tumour-bearing rats in Groups I-a and II-a were used for *in vivo* experiments. Four weeks after the tumours were first detected, the tumour-bearing rats were oophorectomised. One weekly after OVX, each rat was given a single subcutaneous dose of oestradiol (10 µg in 10% ethanol solution/100 g body weight). DMBA-induced tumours were removed under ether anesthesia at 4 h intervals up to 20 h after oestradiol administration. The plasminogen activator activity in each DMBA-induced tumour was assayed as described above.

In vitro *experiments*

Primary cultures of DMBA-mammary tumour cells were initiated and maintained as described previously (Yamashita *et al.*, 1984). When the cells were nearly confluent, the culture medium in each flask was replaced with a serum-free medium by washing with RPMI medium. Each culture was then incubated further for 3 days at 37°C in the serum-free medium prior to adding either oestradiol or the vehicle. Oestradiol dissolved in 0.1% ethanol was added to yield a final concentration of $10^{-8}$ M. Aliquots of culture medium taken at the indicated times were centrifuged at 800 *g* for 10 min at 4°C. The resulting supernatants were assayed for plasminogen activator activity as described above.

*Assay for plasminogen activator inhibitor (PAI) activity*

To clarify whether or not PAI are involved in the hormonal regulation of plasminogen activator in DMBA-tumours, PAI acivity was determined by titrating the tumour extracts or the conditioned medium with human melanoma t-PA or human urokinase and then determining by spectrophotometry the residual plasminogen activator activity, as described (Verheien *et al.*, 1984). The PAI activity was negligible in each tumour examined in the *in vivo* experiments and in every supernatant in the *in vitro* experiments. This suggests that plasminogen activator activity in these samples was modulated directly by a hormone.

*Statistical analysis*

The statistical significance of any difference in plasminogen activator activity and oestrogen receptor content was determined by Student's *t* test. The $\chi^2$ test was used to assess the statistical significance of differences in the incidence or the % diploidy of DMBA-induced mammary tumours in each group.

### Results

*Effects of sex steroids on DMBA-induced mammary tumours*

Three types of palpable mass were found in the mammary glands: mammary carcinomas, grossly visible cysts, and mammary dysplasia (regarded to be a benign lesion). The cystic nodules were soft and filled with a milky fluid, and were identified microscopically as epithelial cysts. Mammary dysplasia was characterised by heterogenous microscopic features including adenosis, fibrosis, duct papillomatosis and fibroadenoma-like lesions. It was difficult to distinguish by palpation mammary carcinomas from nodules. The most common type of mammary dysplasia is adenosis. Therefore, we used this histological type of dysplasia in both *in vivo* and *in vitro* experiments.

The effects of hormonal manipulation on DMBA-induced mammary tumours in rats are summarised in Table I. When 20 mg of DMBA was fed to the NI rats (Group I-a), 100% of the animals developed mammary carcinomas within 50–120 days, and oophorectomy performed simultaneously with DMBA administration prevented completely tumour induction (Group I-b).

Mammary tumours with the microscopic characteristics of dysplasia were induced in 88.9% of the NA rats (Group II-a) and the development of palpable carcinomas in this group was completely suppressed. In NA rats with OVX + P (Groups II-d and II-f) and with OVX + E + P (Group II-e), the number of rats with palpable carcinomas increased significantly ($P<0.001$), as compared to NA rats (Groups II-a), NA rats with OVX (Group II-b), or NA rats with OVX + E (Group II-c). Furthermore, the incidence of DMBA-induced mamary carcinoma was higher in NA rats with P (Groups II-g and II-h) than in NA rats with OVX + P (Groups II-d and II-f) or with OVX + E + P (Group II-e) ($P<0.001$).

*Plasminogen activator activity in DMBA-induced rat mammary tumours*

Table II shows the plasminogen activator activity in DMBA-induced rat mammary tumours in each group. The enzyme activity was significantly higher in DMBA-induced carcinomas than in dysplasia. Lower activity was found in the II-d and II-f carcinoma groups than in the other carcinoma groups.

In vivo *effects of oophorectomy and oestrogen administration on plasminogen activator activity of DMBA-induced rat mammary carcinomas and dysplasia*

As shown in Figure 2, the plasminogen activator activity of DMBA-induced rat mammary carcinomas was under the control of oestradiol *in vivo*. The plasminogen activator activity was 577 units $mg^{-1}$ protein. Within a week after oophorectomy, the activity decreased to less than 7% of baseline (37 units $mg^{-1}$ protein). After administering oestradiol to the oophorectomised tumour-bearing rats, the plasminogen activator activity of the tumours increased to reach a maximum of 376 units $mg^{-1}$ protein after 12 h. This was followed by a gradual decrease to 214 units $mg^{-1}$ protein at 20 h.

DMBA-induced dysplasia displayed significantly lower plasminogen activator activity (68 units $mg^{-1}$ protein) than did DMBA-induced carcinomas. Furthermore, in sharp contrast to the carcinomas, the enzyme activity in dysplasia did change appreciably after oophorectomy or oestradiol administration.

In vitro *effects of oestrogen on plasminogen activator activity in DMBA-induced rat mammary carcinomas and dysplasia*

We examined the effects of oestrogen on plasminogen activator activity in primary cultures of DMBA-induced rat mammary tumour cells in each group. As shown in Figure 3, enzyme activity in the culture medium of group I-a carcinoma cells increased markedly in the presence of $10^{-8}$ M oestradiol, a concentration equivalent to the physiological level.

Furthermore, DMBA-induced rat mammary carcinoma cells in Groups II-e, II-g and II-h showed a similar oestrogen dependency of enzyme production. Since DMBA-induced rat mammary dysplasia (Groups II-a, II-g and II-h) showed no apparent increase in plasminogen activator activity on oestradiol administration, the hormonal regulation of this enzyme was considered to be specific to carcinoma cells. However, an exceptional result was obtained in an *in vitro* experiment with DMBA-induced rat mammary carcinoma cells from Groups II-d and II-f. These cells displayed no appreciable change in plasminogen activator activity, regardless of the presence or absence of oestradiol.

*Oestrogen receptor in DMBA-tumours*

Table III shows the oestrogen receptor content of DMBA-induced rat mammary tumours in each group. The results confirm that each tumour examined in the present study was oestrogen receptor-positive and there were no significant difference in the oestrogen receptor content among the tumours in each group.

### Discussion

There have been only a few reports of the relationship between progesterone and DMBA-mammary carcinogenesis (McCormick & Moon, 1965; Jabara *et al.*, 1973; Nagasawa *et al.*, 1986). Since complicated interactions are observed at the cellular level among several hormones, such as oestrogen, progesterone and prolactin (Nagasawa & Morii, 1981), it is difficult to specify the hormonal environment in the present

**Table I** Effects of sex steroids on induction of DMBA-induced rat mammary tumours

| *Group and treatment*[a] | *No. of rats in group* | *No. of rats with palpable carcinomas (%)*[b] | *No of rats with mammary dysplasia (%)*[b] |
|---|---|---|---|
| I-a: NI[c] | 18 | 18 (100.0) | 0 (0) |
| I-b: NI + OVX[e] | 18 | 0 (0)[k] | 0 (0) |
| II-a: NA[d] | 18 | 0 (0)[k] | 16 (88.9)[k] |
| II-b: NA + OVX[e] | 18 | 0 (0) | 0 (0)[l] |
| II-c: NA + OVX[e] + E[g] | 16 | 0 (0) | 0 (0)[l] |
| II-d: NA + OVX[e] + P[h] | 22 | 6 (27.3)[m,n] | 0 (0)[l] |
| II-e: NA + OVX[e] + E[g] + P[i] | 24 | 5 (20.8) | 0 (0)[l] |
| II-f: NA + OVX[f] + P[i] | 22 | 4 (18.2) | 1 (4.5)[l] |
| II-g: NA + P[i] | 16 | 14 (87.5)[l,o] | 14 (87.5) |
| II-h: NA + P[j] | 16 | 11 (68.8)[l] | 10 (62.5) |

[a]At 50 days of age, 20 mg DMBA was given to rats in every group by gastric intubation. [b]When more than one tumour developed in each animal, all tumours were examined. In the II-g and II-h groups, both carcinoma and dysplasia co-existed in several rats. [c]NI = Neonatally Intact Rats. [d]NA = Neonatally Androgenised rats; 1.25 mg testosterone propionate was given by s.c. injection at 2 days of age. [e]OVX was performed at the same time as DMBA administration. [f]OVX was performed at 28 days after DMBA administration. [g]10 µg E (oestradiol) was given daily by i.m. injection starting from 28 days after DMBA administration. [h]4 mg P (progesterone) was given daily by i.m. injection starting 14 days before DMBA administration. [i]4 mg P was given daily by i.m. injection starting 28 days after DMBA administration. [j]4 mg P was given daily by i.m. injection from 14 days before DMBA administration until the day of DMBA administration. [k]Differs from I-a; $P<0.001$. [l]Differs from II-a; $P<0.001$. [m]Differs from II-a; $P<0.05$. [n]Differs from II-b; $P<0.05$. [o]Differs from II-f; $P<0.001$.

**Table II** Plasminogen activator activity in DMBA-induced rat mammary tumours

| | *Group* | | | | | | | | |
|---|---|---|---|---|---|---|---|---|---|
| | *Dysplasia* | | | *Carcinomas* | | | | | |
| | *II-a* | *II-g* | *II-h* | *I-a* | *II-d* | *II-e* | *II-f* | *II-g* | *II-h* |
| PA activity[a] (mean ± s.d.) | 68 ± 20[b,c] (12) | 62 ± 24[c] (10) | 70 ± 23[c] (10) | 577 ± 128 (12) | 251 ± 73[d] (10) | 641 ± 170 (8) | 198 ± 48[d] (8) | 548 ± 144 (10) | 517 ± 128 (10) |

[a]PA activity; plasminogen activator activity (units $mg^{-1}$ protein). [b]Numbers in parentheses are the numbers of tumours examined. [c]Differs from carcinoma groups; $P<0.001$; [d]Differs from the other carcinoma groups; $P<0.001$.

**Figure 2** *In vivo* effects of oophorectomy and subsequent oestradiol administration on plasminogen activator activity in DMBA-tumours (●, mammary carcinomas in Group I-a; ○, mammary dysplasia in Group II-a). One week after oophorectomy, each DMBA-tumour bearing rat was given by subcutaneous injection a single dose of oestradiol (10 µg/100 g body weight). Tumours were removed at 4 h intervals up to 20 h and assayed for plasminogen activator activity as described under Materials and methods. Solid bars indicate the standard deviation of plasminogen activator activity and n denotes the number of animals tested. During the first week following oophorectomy, 28 mammary carcinoma tumours remained static and 14 of these regressed slightly, while all 42 mammary dysplasia tumours remained static.

**Figure 3** *In vitro* effects of oestrogen on plasminogen activator production. Cultures of DMBA-induced mammary carcinoma cells (Groups I-a, II-d, II-e, II-f, II-g and II-h) and mammary dysplasia cells (Groups II-a, II-g and II-h) were plated in 25-cm$^2$ T-flasks and then incubated for 3 days in a serum-free medium prior to the experiments. After adding $10^{-8}$ M oestradiol (●) or the vehicle (○), aliquots of the culture medium were withdrawn at the indicated times and assayed for plasminogen activator activity as described under Materials and methods. Each point represents the mean value of four individual experiments.

experimental rats. According to earlier reports, however, the levels of serum prolactin and oestrogen in NA rats seem to be within normal range and similar to that in NI rats. The serum progesterone level is, however, much lower in NA rats, all of the corpora lutea being absent in the ovaries, than in NI rats (Yoshida *et al.*, 1980; Cristakos *et al.*, 1976). The results obtained in the present investigation show that supplementation with progesterone in NA rats increases significantly the incidence of DMBA-induced rat mammary carcinoma. This effect is enhanced further by the co-administration of oestrogen (Table I). Yoshida *et al.* (1978, 1980) found, in a detailed study using NA rats, that progesterone promotes mammary carcinogenesis during the growth phase of dysplastic mammary cells. Their experiment was, however, different from ours in the time of oophorectomy and the timing of sex steroid administration. In the present study, we obtained similar results, indicating that progesterone stimulates the growth of DMBA-mammary carcinomas which have already been induced.

Plasminogen activators are serine proteases which convert the inactive zymogen, plasminogen, to the protease, plasmin, a principal enzyme involved in fibrinolysis. Since plasminogen activators are produced by a wide variety of tumour cells, several authors have suggested that the activators can be used as markers of malignant change (Howett *et al.*, 1978; Unkeless *et al.*, 1975; Duffy & O'Grady, 1984; Soumendra & Rajeswari, 1987; Butler *et al.*, 1986). Our *in vivo* and *in vitro* experiments demonstrate clearly that plasminogen activator activity in DMBA-induced rat mammary carcinomas is controlled by oestrogen. In contrast, oestrogen induction of this enzyme was not observed in DMBA-induced rat mammary dysplasia. We previously reported that oestrogen does not have any effect on plasminogen activator activity in either the normal rat uterus or normal breast tissue (Inada *et al.*, 1991) but our present data comprise the first evidence that the

**Table III** Oestrogen receptor contents of DMBA-induced rat mammary tumours

| | Group | *Oestrogen receptor content fmol mg$^{-1}$ protein (mean ± s.d.)* |
|---|---|---|
| Dysplasia | II-a | 27.6 ± 16.9 (7)[a] |
| | II-g | 31.3 ± 18.3 (7) |
| | II-h | 28.2 ± 17.5 (7) |
| Carcinomas | I-a | 43.4 ± 22.0 (7) |
| | II-d | 27.4 ± 15.9 (5) |
| | II-e | 40.0 ± 28.0 (5) |
| | II-f | 28.8 ± 18.1 (5) |
| | II-g | 40.2 ± 24.0 (7) |
| | II-h | 24.2 ± 12.1 (7) |

[a]Numbers in parentheses are the numbers of tumours examined. There is no statistically significant difference in the oestrogen receptor contents of DMBA-tumours among the groups.

oestrogen dependence of plasminogen activator is acquired during the process of malignant transformation of rat mammary cells.

Conflicting data on the oestrogen dependence of the enzyme were obtained for Groups II-d and II-f carcinoma cells *in vitro*. Primary cultures of these carcinoma cells did not exhibit an oestraogen-dependent increase in plasminogen activator. Although every DMBA-induced rat mammary carcinomas examined in the present study was confirmed to be oestrogen receptor-positive, with similar contents, the receptors of the mammary carcinoma cells in Groups II-d and II-f are considered to be non-functional. One explanation would be that an intact ovary is required to see oestrogen effects in culture and oestrogen preconditioning *in vivo* is required to get *in vitro* effects. Thus, these findings support the possibility that during carcinogenesis a hormonal difference contributes to the difference seen in the biologic characteristics of mammary carcinomas, such as the oestrogen dependency of plasminogen activator expression. To our knowledge, there has been no report to date of any significant relationship between hormonal control of plasminogen activator in DMBA-induced rat mammary tumours and the hormonal milieu during neoplastic transformation.

DMBA-mammary tumours are prolactin dependent. High plasma levels of this hormone favor the development and subsequent growth of a mammary tumour, even in oophorectomised rats. A similar phenomenon observed upon the administration of oestrogen may be due to its stimulating prolactin secretion by the pituitary. This view was also supported by the finding that oestrogen has little effect on the growth of DMBA-mammary tumours in hypophysectomised rats. However, data which support the direct involvement of oestrogen are also available, in that in rats bearing DMBA-mammary tumour and in which the hypothalamus had been destroyed, oophorectomy results in a rapid regression of tumours even while a high plasma levels of prolactin is maintained. It is difficult to specify whether in the present study the *in vivo* oestrogen dependency of DMBA-carcinoma is direct or indirect. However, the results of the *in vitro* studies of DMBA-induced carcinoma cells in primary culture (I-a, II-e, II-g and II-h) suggest strongly that oestrogen exerts its function directly. Taken together, our present data indicate that the hormonal milieu dictates the DMBA-induced tumour's hormone sensitivity and that in some of the resulting tumours, oestrogen exerts its effect directly by a route distinct from the prolactin pathway.

This work was supported in part by a Grant-in-Aid for Scientific Research (58570519) from the Ministry of Education, Science and Culture of Japan.

We thank Dr N.J. Halewood and Dr Tetsuro Yamamoto for their valuable advice and discussions.

### References

BRADFORD, M.M. (1976). A rapid and sensitive method for the quantitation of microgram quantities of protein utilizing the principle of protein-dye binding. *Anal. Biochem.*, **72**, 248.

BUTLER, W.B., BERLINSKI, P.J., HILLMAN, R.M., KELSEY, W.H. & TOENNIGES, M.M. (1986). Relation of *in vitro* properties to tumorigenicity for a series of sublines of the human breast cancer cell line MCF-7. *Cancer Res.*, **46**, 6339.

CRISTAKOS, S., SINHA, D. & DAO, T.L. (1976). Neonatal modification on endocrine function and mamary carcinogenesis in the rat. *Br. J. Cancer*, **34**, 58.

DUFFY, M.J. & O'GRADY, P. (1984). Plasminogen activator and cancer: perspectives and commentaries. *Eur. J. Cancer Clin. Oncol.*, **20**, 577.

GREENE, G.L. & JENSEN, E.V. (1982). Monoclonal antibodies as probes for estrogen receptor detection and characterization. *J. Steroid Biochem.*, **16**, 353.

HOWETT, M.K., HIGH, C.S. & RAPP, F. (1978). Production of plasminogen activator by cells transformed by herpes viruses. *Cancer Res.*, **38**, 1075.

INADA, K., YAMASHITA, J., YOSHIMURA, T. & 4 others (1991). Hormonal regulation of plasminogen activator and peroxidase activities in 7,12-dimethylbenz[a]anthracene induced rat mammary tumors and the rat uterus. *Jpn. J. Surg.*, **21**, 249.

JABARA, A.G., TOYNE, P.H. & HARCOURT, A.G. (1973). Effects of time and duration of progesterone administration on mammary tumors induced by 7,12-dimethylbenz[a]anthracene in Sprague-Dawley rats. *Br. J. Cancer*, **27**, 63.

KING, R.J., COWAN, D.M. & INMAN, D.R. (1965). The uptake of [6,7-$^{3}$H] oestradiol by dimethylbenzanthracene-induced rat mammary tumors. *J. Endocrinol.*, **32**, 83.

MCCORMICK, G.M. & MOON, R.C. (1965). Effect of pregnancy and lactation on growth of mammary tumors induced by 7,12-dimethylbenz[a]anthracene (DMBA). *Br. J. Cancer*, **19**, 160.

MOBBS, B.G. (1966). The uptake of tritiated oestradiol by dimethylbenzanthracene-induced mammary tumors of the rat. *J. Endocrinol.*, **36**, 409.

NAGASAWA, H. & MORII, S. (1981). Prophylaxis of spontaneous mammary tumorigenesis by temporal inhibition of prolactin secretion in rats at young ages. *Cancer Res.*, **41**, 1935.

NAGASAWA, H., OHTA, K., NAKAJIMA, K. & 4 others (1986). Interrelationship between pituitary and ovarian hormones in normal and neoplastic mammary growth in mice. *Ann. NY Acad. Sci.*, **464**, 301.

SHIMADA, H., MORI, T., TAKADA, A. & 5 others (1981). Use of chromogenic substrate S-2251 for determination of plasminogen activator in rat ovaries. *Thromb. Haemostas.*, **46**, 507.

SOUMENDRA, N.G. & RAJESWARI, S. (1987). Expression of tumor cell properties in synovial cells in culture. *Acta Cytologica*, **31**, 77.

THORSEN, T. (1982). Association of plasminogen activator activity and steroid receptors in human breast cancer. *Eur. J. Cancer Clin. Oncol.*, **18**, 129.

UNKELESS, J.C., TOBIA, A., QUIGLEY, J.P., RIFKIN, D.B. & REICH, E. (1975). An enzymatic function associated with transformation of fibroblasts by oncogenic viruses. I. Chick embryo fibroblast cultures transformed by avian RNA tumor viruses. *J. Exp. Med.*, **137**, 85.

YAMASHITA, J., HORIUCHI, S., SHIGAKI, N., FUJINO, N. & AKAGI, M. (1984). Estrogen-dependent plasminogen activator in 7,12-dimethylbenz[a]anthracene-induced rat mammary tumors *in vivo* and *in vitro*. *Gann*, **75**, 681.

YAMASHITA, J., HORIUCHI, S., KIMURA, M., NISHIMURA, R. & AKAGI, M. (1986). Plasminogen activator as a functional marker for estrogen dependence in human breast cancer cells. *Jpn. J. Cancer Res.*, **77**, 177.

YOSHIDA, H. & FUKUNISHI, R. (1978). Effect of neonatal administration of sex steroids on 7,12-dimethylbenz[a]anthracene mammary carcinoma and dysplasia in female Sprague-Dawley rats. *Gann*, **69**, 627.

YOSHIDA, H., FUKUNISHI, R., KATO, Y. & MATSUMOTO, K. (1980). Progesterone-stimulated growth of mammary carcinomas induced by 7,12-dimethylbenz[a]anthracene in neonatally androgenized rats. *J. Natl Cancer Inst.*, **65**, 823.

VERHEIJEN, J.H., CHANG, G.T.G. & KLUFT, C. (1984). Evidence for the occurrence of fast-acting inhibitor for tissue-type plasminogen activator in human plasma. *Thromb. Haemostas.*, **51**, 392.